# Anti-Inflammatory and Antioxidant Activity of *Litsea glaucescens* Kunth in Rodents, an Aztec Medicinal Plant Used in Pre-Columbian Times

**DOI:** 10.3390/ph19010040

**Published:** 2025-12-23

**Authors:** Dulce Yehimi López-Miranda, Ricardo Reyes-Chilpa, Antonio Nieto-Camacho, Silvia Laura Guzmán-Gutiérrez, Oscar Salvador Barrera-Vázquez, María Sofía Jiménez-Mendoza, Eréndira García-Ríos, Gil Alfonso Magos-Guerrero

**Affiliations:** 1 Facultad de Medicina, Universidad Nacional Autónoma de México, Ciudad Universitaria, Mexico City 04510, Mexico; yehimilm12@ciencias.unam.mx (D.Y.L.-M.); osbarrerav@comunidad.unam.mx (O.S.B.-V.); 2 Posgrado en Ciencias Biológicas, Universidad Nacional Autónoma de México, Mexico City 04510, Mexico; mariasofia009@ciencias.unam.mx; 3 Instituto de Química, Universidad Nacional Autónoma de México, Ciudad Universitaria, Mexico City 04510, Mexico; camanico2015@gmail.com (A.N.-C.); erengr@unam.mx (E.G.-R.); 4 Casa Libertad, Colegio de Ciencias y Humanidades, Universidad Autónoma de la Ciudad de México, Mexico City 09620, Mexico; laura.guzman.gutierrez@uacm.edu.mx

**Keywords:** *Litsea glaucescens*, *Litsea guatemalensis*, anti-inflammatory, Codex de la Cruz-Badiano

## Abstract

**Background/Objectives:** *Litsea glaucescens* Kunth, commonly known as “laurel,” is a tree native to Mexico. The Codex Cruz-Badiano, from 1552, described it as the main ingredient of a topical anti-inflammatory recipe. This study aims to determine whether *L. glaucescens* leaf extract can reduce experimental inflammation, supporting its use in Aztec medicine. **Methods**: Methanolic extracts and fractions from the leaves of *L. glaucescens* were analyzed using techniques such as normal and reverse-phase TLC, 1H-NMR, HPLC-UV, MS, and GC-MS. The anti-inflammatory systemic activity of this methanolic extract was evaluated in mice using carrageenan-induced paw inflammation and TPA-induced ear topical inflammation models. Myeloperoxidase activity, DPPH, and TBARS assays were performed. *L. guatemalensis*, a closely related species, served as a positive control, as its biological activity has been demonstrated. **Results**: Thin-layer chromatography analysis reveals flavonoid-type compounds in the methanolic extract of *L. glaucescens* leaves, and when it was fractionated, pinocembrin and quercitrin were the main compounds found. *L. glaucescens* in mice significantly reduced carrageenan-induced paw swelling and TPA-induced ear inflammation. A decrease in myeloperoxidase activity and an increase in antioxidant activity were observed. **Conclusions**: Methanolic extract from *L. glaucescens*, administered systematically, produced significant in vivo anti-edematous effects and in vitro, antioxidant and anti-infiltrative/anti-neutrophilic activities, qualitatively like those of *L. guatemalensis*. Quercitrin and pinocembrin could contribute to these actions. It is unclear which of the two plant species was used in pre-Columbian times; However, our results show that both species contain phytochemicals with anti-inflammatory properties, suggesting that the Aztecs recognized this medicinal property.

## 1. Introduction

**Figure 1:**
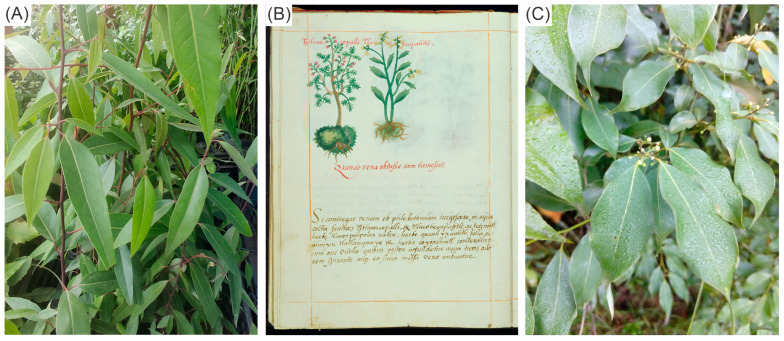
(**A**) *L. guatemalensis* from Los Sauces, Morelos, Mexico. (**B**) *Folium 48v* of the Codex de la Cruz-Badiano with the drawings of *tzihuac copalli* (*Bursera bipinnata*, Burseraceae), and “*tlacoecapahtli*” (*L. glaucescens*, Lauraceae). (**C**) *L. glaucescens* de Zacatlán, Puebla, México. Photographs by the first author.

*Litsea glaucescens* Kunth, also known as laurel in Mexico, is one of eight American *Litsea* tree species, seven of which thrive in Mexico. Variations in leaf morphology and pubescence can differentiate these species [1]. *L. glaucescens* is classified as endangered by the Secretariat of Environment and Natural Resources of Mexico (SEMARNAT) [2]. Despite their status, laurel leaves are among the country’s main non-timber forest products due to their diverse uses [3]. The most important being as a condiment [4], followed by medicinal [5] and religious uses [6]. The medicinal properties of *L. glaucescens* are first mentioned in the Cruz-Badiano Codex, really entitled “*Libellus de Medicinalibus Indorum Herbis*” (Little Book of Medicinal Herbs of the Indies). It was dictated in Nahuatl and translated into Latin in 1552 by the native erudite Martín de la Cruz (physician) and Juan Badiano (lecturer), respectively, at the Colegio de la Santa Cruz de Tlatelolco, which was devoted to the education of the indigenous noblemen’s children [7]. In *Folium 48v* of the Codex, a plant remedy is described that is recommended for treating swelling from punctured veins as a consequence of ancient phlebotomy practices [8]. The remedy calls for grinding and cooking buds of “*tzihuac copalli*” (*Bursera bipinnata*, Burseraceae), “*tetzmitl*” (*Sedum dendroideum*, Crassulaceae), and “*tlacoecapahtli*” (*Litsea glaucescens*, Lauraceae) with water, adding egg yolk, and mixing everything with water that smells like incense. The juice is spread to the swollen vein [8]. Only the first two plants were drawn (Figure 1). It has been proposed from the “*tlacoecapahtli*” drawing that it is *L. glaucescens* [9]; however, as further discussed, it could be the related species *L. guatemalensis*.

Based on historical documents, such as codices [8,10,11] and chronicles [12,13,14], the Aztecs performed bloodletting with thorns and obsidian blades. This procedure was usually performed by experts associated with religion and sacrifice. Bloodletting was also employed in certain instances to treat medical conditions, including hormonal imbalances, wounds, and headaches [15]. In 1521, following Spain’s assumption of control, phlebotomy was introduced in Mexico, then known as New Spain. This method, originating in Europe, was used to treat various illnesses, including swelling. In New Spain, bloodletting was common but often led to infections due to poor hygiene. The Mexica (Aztec) culture is recognized for its extensive history of using herbal remedies to address a range of inflammatory ailments; thus, the widespread application of plant-based treatments for phlebitis is unsurprising. Over time, the use of medicinal plants for treating phlebitis was abandoned, primarily because phlebotomy was determined to be an ineffective treatment. Louis Pasteur (1822–1895) and Robert Koch (1843–1910) demonstrated that inflammation results from an infectious process [16].

**Figure 2:**
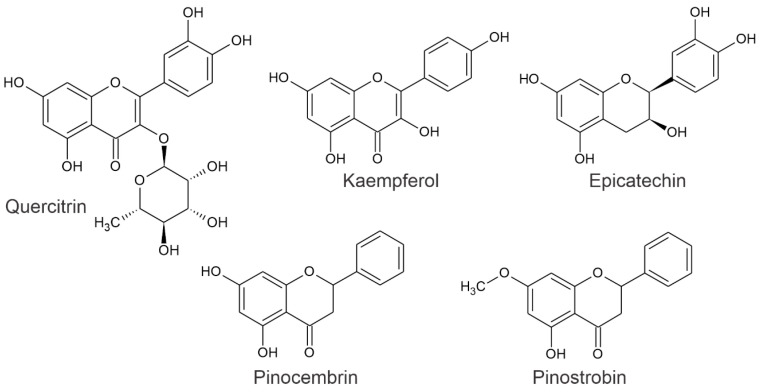
Identified flavonoids from *L. glaucescens*.

*Bursera bipinnata* and *Sedum dendroidum*, included in the original recipe of the Cruz-Badiano Codex (*Folium 48v*), are still used in Mexican folk medicine for their anti-inflammatory effects [8]. This underscores the Aztecs’ strong recognition of the medicinal properties of plants. *L. glaucescens* is no longer used because there is no record of its use in treating inflammation, despite its mention in the codex. It is worth noting that *Bursera bipinnata* [17] and *Sedum dendroideum* [18] have shown anti-inflammatory activity. Therefore, we can hypothesize that *L. glaucescens* could also exhibit similar properties. This possibility increases because the leaves of *L. glaucescens* contain quercetin, epicatechin, pinocembrin, pinostrobin, and kaempferol [19,20]. Figure 2 illustrates the main active substances identified in *L. glaucescens*. Research has shown that pinocembrin and pinostrobin reduced swelling in HCF-1 gum cells exposed to silver nanoparticles (AgNPs) [21]. Quercetin (10 mg/kg) reduced inflammation in rats with carrageenan-induced inflammation, as shown by decreased levels of PGE2, TNF-α, and COX-2 mRNA [22]. On the other hand, the leaves of *L. guatemalensis*, a species closely related to *L. glaucescens* due to its chemical content, reduced inflammation in mice when tested with carrageenan and exhibited anti-hyperalgesic effects, as evaluated using the mouse paw formalin model [23]. In addition, this species, as will be discussed, might be the focus of the Cruz-Badiano Codex illustration (*Folium 48v*). Given the questionable evidence concerning the plant species referenced in the codex and the necessity to correlate the ethnomedicinal applications of these *Litsea* spp. with contemporary scientific data, we opted to investigate the preclinical anti-inflammatory effects and visualize someone of the components of methanolic extracts derived from *L. glaucescens* and of *L. guatemalensis* to evaluate whether the identification of the anti-inflammatory effect by the pre-Columbian culture in Mexico can be initially explored in experimental models of inflammation.

## 2. Results

### 2.1. Thin-Layer Chromatography (TLC) of Extracts and Fractions

Following methanol maceration of the dried leaves of each *Litsea* spp., the extract yield was 24.76% for *L. glaucescens* (GLAM) and 17.30% for *L. guatemalensis* (GUAM). Hexane washing of 23 g of GLAM extract created two parts: GLAMF (15.82 g), which dissolves in methanol, and GLAHF (6.82 g), which dissolves in hexane. In the derivatization with 1% of 2-aminoethyl diphenylborinate, yellow zones were observed in TLC analysis, indicating flavonoids in the complete extracts of GLAM and GUAM and in the fractions GLAMF and GUAMF. More yellow zones are revealed in *L. guatemalensis* (Figure S1).

### 2.2. HPLC Analysis of GLAM

**Figure 3:**
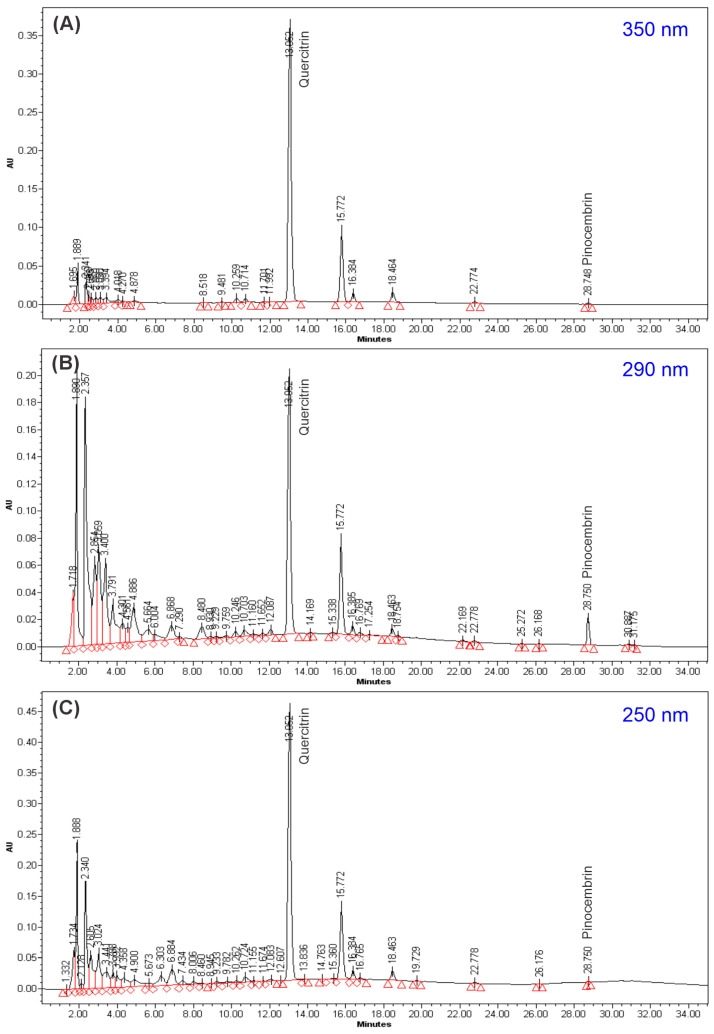
Chromatograms of the *L. glaucescens* methanolic extract (GLAM) at 350 nm (**A**), 290 nm (**B**), and 250 nm (**C**). Wavelengths at which flavonoids usually show absorbance. Quercitrin (RT: 13.066 min) and pinocembrin (RT: 28.763) absorb at these wavelengths.

High-Performance Liquid Chromatography (HPLC) was used to generate the chromatograms shown in Figure 3, which illustrate the chromatographic spectrum of the GLAM extract at 350 nm (A), 250 nm (B), and 290 nm (C). At this wavelength, flavonoids typically exhibit absorbance. The peak with an average retention time (RT) of 13.066 min represents the major component of the GLAM extract, accounting for 60.18% at 350 nm, 17.50% at 290 nm, and 33.96% at 250 nm. This peak was identified as quercitrin (Figure S2) because it exhibited an RT (12.958) and UV spectrum similar to those of the reference standard (Figure S3). The peak with an average RT of 28.763 was identified as pinocembrin, as it exhibited RT (28.835) and absorbance values similar to those of the standard (Figures S2 and S3).

### 2.3. Analysis of GLAMF Fractionation, HPLC, and MS

**Figure 4:**
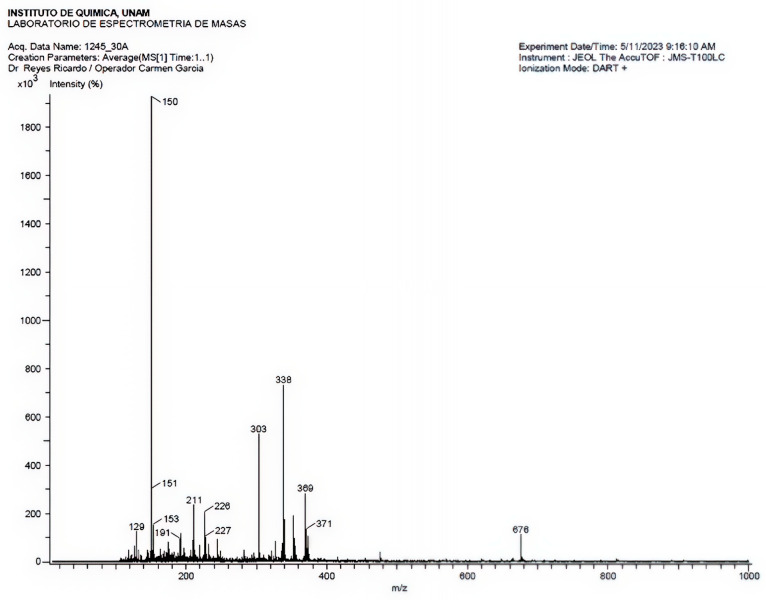
Mass spectra in positive-ion mode of the fraction obtained from a SP cartridge eluted with a water: methanol 70:30 mixture. The fragmentation pattern shows a peak at 303 *m*/*z*, characteristic of the molecular ion of quercetin, and another at 338 *m*/*z*, possibly from dehydrated quercetin (Figure 4).

An SPE cartridge was used to fractionate 50 mg of GLAMF, which was then eluted with a water-methanol mixture. The fraction obtained with a 70:30 water: methanol mixture was analyzed by HPLC-DAD, which showed absorbance of 390 mAU at 340 nm and 210 mAU at 280 nm, with an RT of 4.831 min. The amount collected from this fraction was 4.4 mg (8.8%). We used DART-MS in positive-ion mode to study this fraction. The results showed a peak at *m*/*z* 303, characteristic of the molecular ion of quercetin, and another at *m*/*z* 338, possibly from dehydrated quercetin (Figure 4). The chromatogram of the 70:30 (water:methanol) fraction derived from GLAMF extract fractionation via SP cartridge is presented in Figure S4.

### 2.4. GLAHF Fractionation, GC-MS

**Figure 5:**
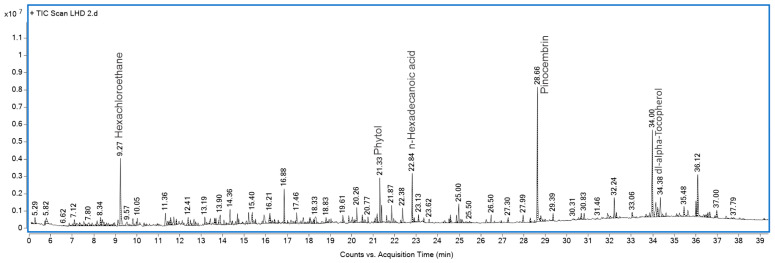
Gas chromatography-mass spectrometry analysis of the hexane fractions from *L. glaucescens*. The compounds were identified using the National Institute of Standards and Technology (NIST) database.

The data obtained from GLAHF by gas chromatography-mass spectrometry (GC-MS) were compared with the National Institute of Standards and Technology (NIST) database, which revealed pinocembrin as the primary compound (17.08%) at RT of 28.66 (Figure 5). This information corroborates the reports of López et al. [24] and López Romero et al. [25]. The peaks at 34.00 and 22.84 were identified as vitamin E and palmitic acid, respectively. Table S1 summarizes the compounds identified in GLAHF using the NIST database. The hexane fraction of *L. glaucescens* shows a significantly elevated percentage of pinocembrin. The compounds were identified using the National Institute of Standards and Technology (NIST) database. Five grams of GLAH were fractionated on a chromatographic column packed with 60 M silica gel and eluted with hexane and mixtures of hexane and ethyl acetate from 100% hexane to 0% hexane and from 0% ethyl acetate to 100% ethyl acetate. One hundred and four 20 mL fractions were obtained. The fractions were analyzed by TLC, and fractions 39–49 exhibited a chromatographic zone with a retention factor (RF) like that of the pinocembrin standard.

### 2.5. 1HRMN Analysis from GLAMF and GLAHF

**Figure 6:**
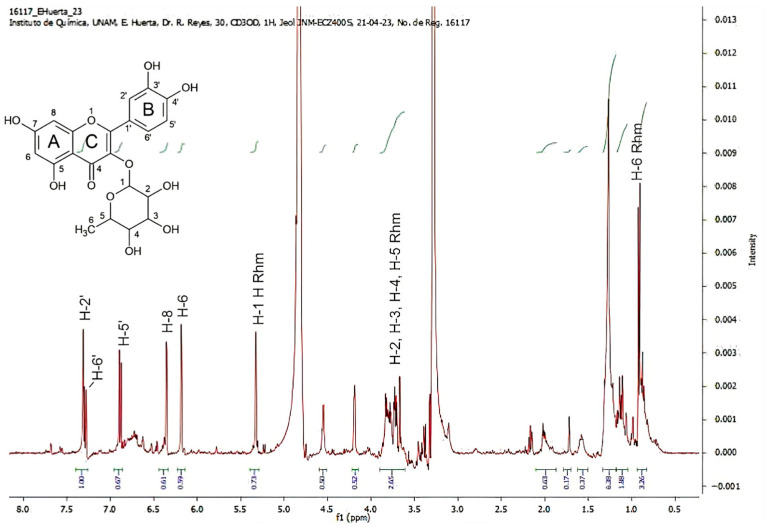
1H RMN of the fraction obtained from an SP cartridge eluted with a mixture of 70% water and 30% methanol. A, B, and C are aromatic rings. The numbers indicate the carbon atoms in the molecule. The signals correspond to quercitrin. The green signals represent integrations indicating the number of hydrogens.

The 1H NMR shows aromatic protons between 6.2 and 7.4 ppm corresponding to the A-B ring, the rhamnose-related signals, 3.5–4.0 ppm, correspond to H-2, H-3, H-4, H-5 in the sugar, the protons of the methyl group in H-6 is observed as a doublet in the aliphatic region at 0.93 ppm, and H-1 is present at 5.3 ppm as a doublet (Figure 6).

**Figure 7:**
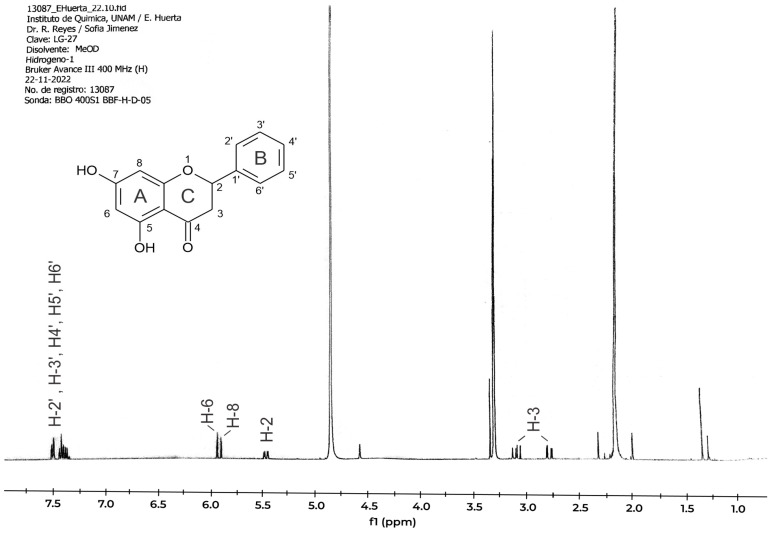
1H RMN of fraction 39 obtained from the fractionation of GLAHF extracts. A, B, and C are aromatic rings. The numbers indicate the carbon atoms in the molecule. The signals correspond to pinocembrin.

Figure 7 shows the 1H NMR spectrum of the sample obtained by fractionation of the complete GLAHF extract. The protons H-2 and H-6 in ring A show up as two peaks (doublets) between 5.8 and 6.0 ppm. The five protons in ring B appear as a group of peaks (multiplet) at 7.6 ppm. In ring C, the proton H-2 is found at 5.5 ppm, while H-3 is seen as a doublet of doublets between 2.75 and 3.20 ppm.

### 2.6. Effects of GLAM and GUAM on Carrageenan-Induced Paw Inflammation

**Figure 8:**
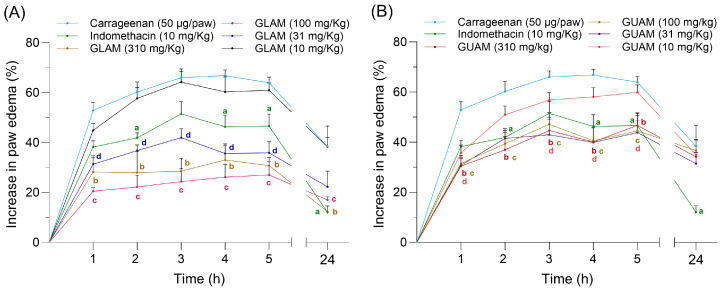
Time evolution of the effects produced by increased doses of (**A**) GLAM and (**B**) GUAM over the increase in paw edema induced by carrageenan in CD1 mice during 24 h. Time course of the effects of various treatments on carrageenan-induced inflammation in the paws of CD1 mice. Statistically significant differences (*p* < 0.05) between carrageenan and treatments are indicated by letters. a: carrageenan vs. indomethacin; b: carrageenan vs. 310 mg (GLAM or GUAM); c: carrageenan vs. 100 mg (GLAM or GUAM); and d: carrageenan vs. 31 mg (GLAM or GUAM).

The average paw thickness (edema) values recorded before, during the first 6 h, and at 24 h after carrageenan injection in mice from the different experimental groups are summarized in Table S2 (GLAM) and Table S3 (GUAM). Sub-plantar administration of carrageenan resulted in an increase in paw thickness in all experimental groups. This increase was greater in the group of animals treated with carrageenan only (Tables S2 and S3). Pretreatment with GLAM and GUAM extracts administered intraperitoneally at doses of 10, 31, 100, and 310 mg/kg (0.1 mL per 10 g of mouse weight) altered, to varying degrees, the intensity of edema at different recording times. The effects of various doses of GLAM and GUAM extracts on the temporal evolution of carrageenan-induced edema intensity are shown in Figure 8A (GLAM) and Figure 8B (GUAM). The paw thickness obtained using the procedure described in the methodology section was significantly lower in mice pre-treated with 31, 100, and 310 mg of GLAM extract than in the control groups pre-treated with saline or indomethacin.

Except for the 10 mg/kg dose, this edema-preventive effect was observed within the first hour across all GLAM extract doses, reaching its maximum at 24 h. In the control group, which received carrageenan and saline, the percentage of inflammation increased to over 60% and remained elevated for the first 5 h, decreasing by 24 h without returning to baseline. The 100 mg/kg dose of GLAM extract produced the most anti-edematous effect, even greater than that of 310 mg/kg. Administration of 10 mg/kg of GLAM extract does not prevent the carrageenan-induced inflammation (Figure 8A). The administered doses of GUAM extract, except the 10 mg/kg dose, also prevented the increase in edema during the 24 h recording period (Figure 8B). The magnitude of the anti-edema effect produced by the GUAM extract is less (around 20%) than the preventive effect of GLAM extract (around 40%) when these effects are compared with the edema produced by carrageenan in the control group. The *p*-values corresponding to each treatment time point to GLAM and GUAM extracts are summarized in Tables S2 and S3, respectively.

### 2.7. Effects of GLAM and GUAM on TPA-Induced Mouse Ear Inflammation

**Figure 9:**
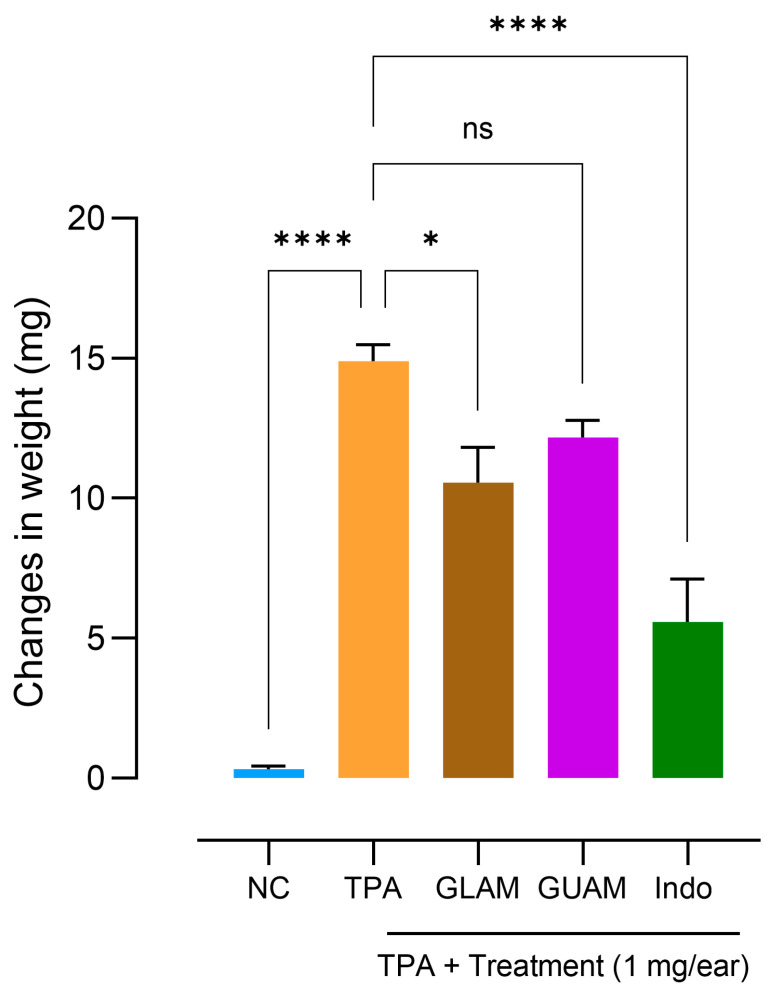
Effect of different treatments on the weight of the right ears treated topically with TPA. The normal control group (NC) represents the weight of the left ears treated with the vehicles without TPA. The asterisks show groups that differ significantly from the TPA in the ethanolic solution group * *p* < 0.05, **** *p* < 0.0005, and ns: non-significant. The bars represent the standard error of the mean, n = 7 animals per group.

Table 1 shows that the left ears without TPA (second column) weighed much less than the right ears with TPA (third column). TPA applied to the right ears resulted in a significant increase in ear weight compared with the left ears (approximately 15 mg). The second column shows the weight of the left ear without TPA, which serves as the normal control (NC) group. Column 1 shows the treatments (Table 1). Figure 9 shows the effect of treatment on the weight of the right ear, measured in mg. The weight difference between the left and right ears, the latter treated with TPA, reflects the weight changes represented on the y-axis. The magnitude of the weight difference indicates the extent of edema in each group. Indomethacin (positive control) prevents the increase in weight (approximately 10 mg) in the right ear when combined with TPA. The weight is derived from a comparison of the maximum increase in weight (15 mg) observed in the TPA-treated group in an alcohol solution (10 µg applied topically). A discrete decrease in the weights of the right ears treated with TPA plus GUAM extract was observed, but it was not significant. In contrast, the GLAM extract reduced right-ear weight, although it was less effective than indomethacin (Figure 9). The Normal Control (NC) group is defined by the vehicle’s impact (ethanol, 10 µL; methanol, 20 µL) on the left ear without TPA. Vehicles do not increase the weight of the left ears (Figure 9). The *p*-values corresponding to the treatment of GLAM and GUAM extracts are summarized in Table S4.

### 2.8. Inhibition of Myeloperoxidase (MPO) Activity

**Figure 10:**
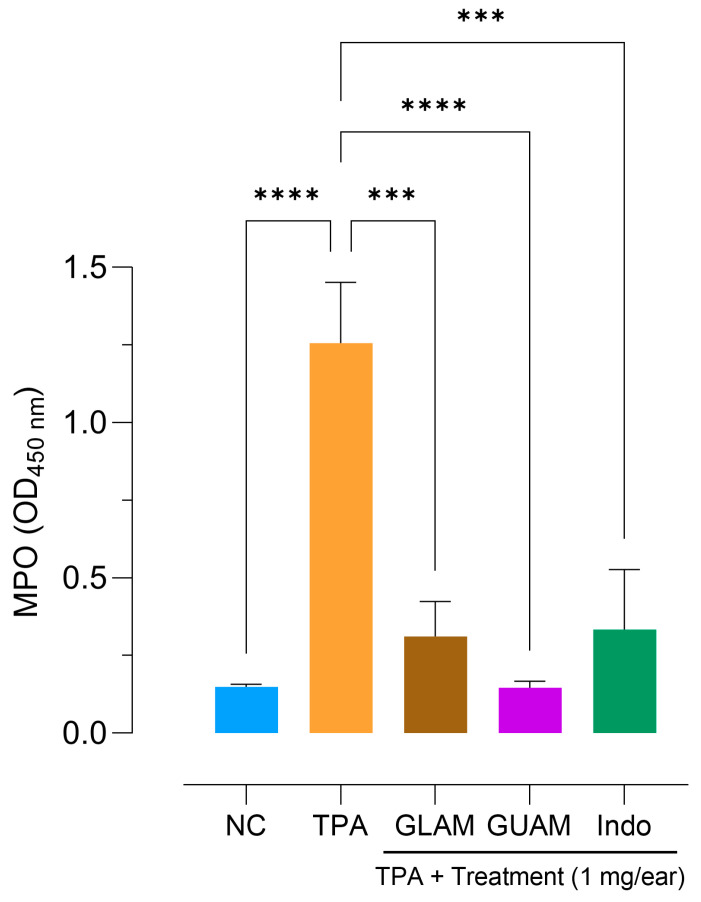
Effect of different treatments on TPA-induced MPO activity in the right ears of the mouse. The magnitude of the activity is calculated by comparing the results of each treatment with those of the TPA group in ethanol solution. The asterisks show groups that differ significantly from the TPA-only group: *** *p* = 0.001, **** *p* < 0.0001. NC: Normal Control; OD = optical density.

As expected, a significant increase in optical density (OD) was observed in the TPA-treated group, reflecting increased myeloperoxidase activity due to neutrophil infiltration in the tissues of the right ear treated with TPA (Table 2). The local application of GLAM and GUAM leaf extracts resulted in marked reductions in MPO activity, with 75.2% and 88.3% efficacy, respectively. Figure 10 shows that the GUAM extract reduced myeloperoxidase activity in the right ear more effectively compared to the GLAM extract and indomethacin. The normal control group (NC), which did not change in MPO activity, received vehicle, ethanol, and methanol in the left ear. The *p*-values corresponding to the treatment of GLAM and GUAM extracts are summarized in Table S5.

### 2.9. Analysis of DPPH Free Radical Scavenging Activity

**Figure 11:**
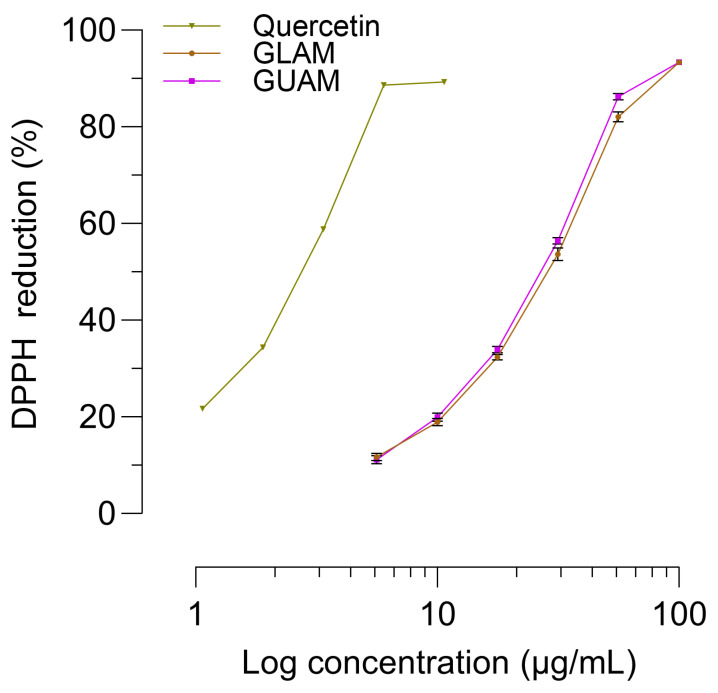
In vitro analysis of the effect of diverse treatments on DPPH reduction activity at various dosages. Symbols show the percentage of DPPH reduction, and the bars represent the SEM: standard error of the mean.

Table 3 presents the effect of GLAM and GUAM leaf extracts on DPPH radical removal in vitro. The decrease in absorbance is significant and directly proportional to the extract’s concentration. As shown by the absorbance values, the efficacy of both extracts for scavenging DPPH radicals is similar (Table 3 and Figure 11). Figure 11 shows the concentration-response curves for the percentage reduction in DPPH, obtained by increasing the extract concentration by a quarter-log unit. The maximum efficacy of both extracts is the same, and their potency, as shown by IC_50_ values, is very close: 27.4 ± 0.74 µg/mL for GLAM and 29.0 ± 1.29 µg/mL for GUAM. Quercitrin, an abundant component of the extract, is slightly less effective than the GLAM and GUAM extracts. As shown in Figure 9, quercetin is more potent than the extracts, as its concentration-response curve shows a leftward shift (IC_50_ = 2.9 ± 0.37 µg/mL). Approximately ten times less quercetin is required to reduce the DPPH radical by 50%.

### 2.10. TBARS Assay Antioxidant Activity

**Figure 12:**
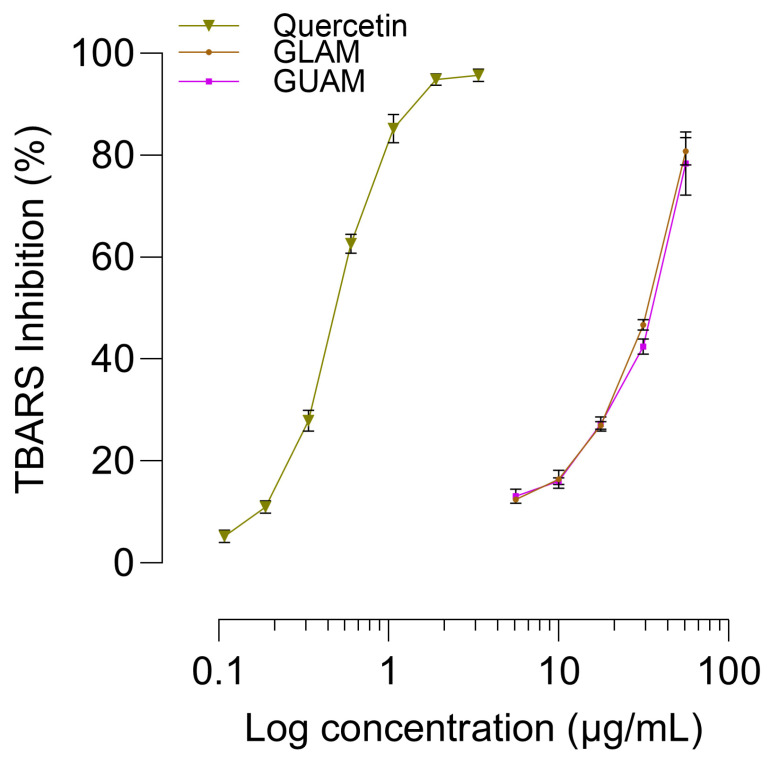
Effect of different treatments on TBARS inhibitory activity in the rat brain. The symbols represent the mean percentage differences in absorbance between the control and treated groups. The bars represent the SEM: Standard error of the mean.

The mean absorbance values for each concentration used in the generation of concentration-response curves with GLAM and GUAM extracts are summarized in Table 4. A notable reduction in nm/mg of protein was noted with increasing doses of each methanolic extract. The decrease is attributable to the amount of methanolic extract used and to its ability to reduce thiobarbituric acid-reactive substances (TBARS) levels, indicating reduced lipid peroxidation and oxidative damage (Figure 12). The percentage reductions in TBARS at the maximum concentrations of GLAM and GUAM were 95.15% and 94.43%, respectively. The efficacy and potency in inhibiting TBARS are very similar between the two extracts (Figure 10). GLAM showed an IC_50_ of 24.43 µg/mL, while GUAM had an IC_50_ of 38.09 µg/mL. In contrast, the concentration-response curve for positive control (quercetin) shows a leftward shift, showing increased potency, with an IC_50_ of 0.506 ± 0.01 µg/mL.

## 3. Discussion

Herbal medicine is part of traditional medicine systems and is recognized for its potential to contribute to health and well-being (OMS). In pre-Hispanic Mexico, indigenous knowledge of medicinal plants as documented in texts written after the Spanish conquest. One notable example is the *Libellus de Medicinalibus Indorum Herbis,* also known as Codex Cruz-Badiano. This manuscript integrates elements of European medicine with the concepts of pre-Hispanic medicine as practiced by Nahua physicians [26]. The work under discussion references 251 plant species and includes 185 illustrations [27]. To undertake the challenging task of approximating their possible botanical identifications, only four elements are available: (1) the illustration itself, (2) the Nahuatl name, (3) geographic distribution, and (4) medicinal properties [26,28]. Among the 185 illustrations in the Codex Cruz-Badiano, Valdés et al. (1992) identified 83 at the species level [28,29].

The interpretation that “*tlaco-ecapahtli”* in *folium 48v* of the Codex Cruz-Badiano as *L. glaucescens* by Bye and Linares (2013) [9] is not definitive, since it is difficult to identify the species from the drawing accurately, and could be a related species, especially *L. guatemalensis*. This species can be recognized by its acuminate leaves, short petioles, and the presence of trichomes on the undersides of its leaves. In contrast, *L. glaucescens* features elliptical leaves and long petioles, and notably lacks trichomes—a key characteristic for distinguishing the two species that is not evident in the illustration. Therefore, the drawing on *folium 48v* could represent either species, since they can coexist in the same areas of central Mexico [1,30,31].

The Aztec people had access to both species and could have used them for the same medicinal purpose. Martin de la Cruz, an indigenous physician at the Imperial College of Indians of Santa Cruz Tlatelolco and the author of the Codex, was not the illustrator [32]. This work was done by indigenous painters and scribes *“tlacuilos”*, who were possibly students of the College [33]. According to current literature, *L. guatemalensis* continues to be used for anti-inflammatory purposes, and preclinical chemical and pharmacological studies support its efficacy [23,34]. In contrast, *L. glaucescens* lacks experimental evidence of an anti-inflammatory effect. For the first time, we provide evidence that a methanolic extract of *L. glaucescens* reduces inflammation when applied topically or systemically.

In the carrageenan model, the anti-inflammatory effect following systemic administration of each extract exhibits qualitative similarity but quantitative disparity, with the GLAM extract showing a significantly more pronounced effect across all assessment points compared to the GUAM extract and indomethacin. The extracts might have similar effects due to their shared anti-inflammatory ingredients [35,36,37,38]. The magnitude of the effects could also be modulated by additional compounds in the extracts, as observed in other anti-inflammatory plants [39]. An in silico study of the phytochemicals of *L. guatemalensis* shows that scopoletin, pinocembrin, and 5,7,3′,4′-Tetrahydroxy-isoflavone could have anti-inflammatory properties through the JAK system (Janus Kinase) JAK1, JAK2, and JAK3 [40]. This signaling system has been reported to be involved in early events of inflammation, such as (1) cytokine signaling, including cytokines and interferons, many of which are key pro-inflammatory mediators released in the early stages of an immune response, such as IL-6, IFN-γ, and TNF-α [41]. (2) Immune cell recruitment and activation, in which, during the initial inflammatory phase, resident immune cells like macrophages and mast cells are activated and release inflammatory mediators. These signals also recruit other immune cells, such as neutrophils [42], and (3) Modulating Phenotypes: The specific JAKs activated influence the polarization of immune cells (such as macrophages and T cells) towards either pro-inflammatory or anti-inflammatory phenotypes. These potential targets may also contribute to the anti-inflammatory effects of constituents from *L. glaucescens.* Since similar chemicals are found in several *Litsea* spp., they may share anti-inflammatory effects through the same targets [34,43].

The results obtained with both *Litsea* spp., the edema formation, and TPA-induced inhibition of MPO activity in the mouse ear, are paradoxical. Although TPA-induced MPO activity was significantly reduced by GLAM, GUAM, and indomethacin (Figure 6), these treatments modestly affected the edema induced by TPA (Figure 7). This paradox, where we obtained little effect on edema and a large effect on MPO, could involve the following possible scenarios, according to previous reports: (1) There are several mechanisms of both inflammatory markers (edema and MPO activity) [44]. Edema typically peaks early (around 6 h), a response primarily driven by initial vascular changes and the release of early inflammatory mediators such as histamine, serotonin, and prostaglandins [45]. In contrast, MPO activity, a marker of neutrophil accumulation, peaks much later (approximately 24 h), as neutrophils require time to infiltrate the tissue [42] entirely. (2) Distinct mechanisms, due to edema, involve increased capillary permeability and fluid accumulation in the tissues, mediated by initial inflammatory signals that cause vasodilation [46,47]. Moreover, MPO activity is directly correlated with the number of neutrophils in the tissue, reflecting a later stage of cellular infiltration in the inflammatory response [44,48].

In this sense, in conjunction with previous reports, a pharmacological agent or condition may activate one pathway more than the other or be administered at a time point that affects one peak but not the other. Research suggests that while COX and LOX inhibitors are effective at reducing MPO levels, their impact on edema is limited. This scenario proposes that lipid mediators may affect neutrophil recruitment (as measured by MPO activity) more than they affect edema fluid accumulation. This finding underscores the sensitivity of MPO accumulation to external factors and proposes that the edema response may operate independently. This evidence may help explain the lack of correlation between edema and MPO activity, suggesting that they are separate indicators of the complex inflammatory process, reflecting different phases and mediator pathways [44,48].

Quercitrin is the abundant active constituent found in the methanolic extract of *L. glaucescens* (GLAMF). According to its structure, quercitrin is quercetin attached to a sugar (rhamnose), making it a glycoside. In the body, intestinal bacteria can break down quercitrin, releasing quercetin (38), which is known for its multiple effects, including the anti-inflammatory effects that occur through various mechanisms, including: (1) Antioxidant effect [49]. (2) Reducing the expression and secretion of inflammatory cytokines and chemokines [50,51]. (3) Down-regulating the NF-κB pathway [52], which has been activated after the sub-plantar injection of carrageenan to induce inflammation and TPA administration [53,54]. In the consulted literature, quercitrin has not been reported in *L. guatemalensis*, and differences in plant yield may explain the lower intensity and duration of the effect observed with the GUAM extract in the carrageenan-induced inflammation model. Pinocembrin, an abundant compound found in the less polar fraction of extract GLAHF leaves, has been reported in *L. glaucescens* [23] and *L. guatemalensis* [23]. Their anti-inflammatory activity has been explained by two primary mechanisms: 1. Inhibition of the TLR4-NF-κB-NLRP3 inflammasome pathway, and 2. Reduction in Th2 cytokines, specifically interleukins (IL)-4, IL-5, and IL-13 [55]. Many of these mediators are also involved in TPA and carrageenan-induced acute inflammation [53,54]. Treatment with pinocembrin significantly lowered the levels of these Th2 cytokines in bronchoalveolar lavage fluid and reduced OVAlbumin-specific immunoglobulin E (IgE) in serum [56]. NF-κB activation induced by carrageenan and TPA is essential for Th2 cytokine and IgE production. Based on our research and existing studies on the effects of quercitrin and pinocembrin on inflammation, we consider that these compounds may contribute to the anti-inflammatory properties of *L. glaucescens* leaves. However, further experimental validation is required. Nearly 473 years after the Codex de la Cruz-Badiano was written, there is a frail connection between the pre-Columbian recommendations and the current use of most of the plants described [57]. In this context, regarding *L. glaucescens* or *L. guatemalensis*, the swelling of the punctured vein mentioned in the Codex is no longer observed today. As a result, the plants traditionally used to ease this complication of venipuncture have fallen out of use. However, local literature on inflammation indicates that branches of *L. glaucescens*, with or without flowers, are boiled with three other plants to prepare a traditional Mexican medicine used in postpartum baths. This practice aims to prevent postpartum swelling in women [58]. The current application of traditional practices reflects the dynamic nature of traditional Mexican medicine, which adapts to changing socio-cultural circumstances and the emergence of modern diseases such as hypertension, obesity, and diabetes [57].

Our results provide preliminary scientific evidence that both *L. glaucescens* and *L. guatemalensis* exhibit in vitro antioxidant and anti-infiltrative/anti-neutrophilic activities, as well as moderate anti-edematous activity, which was not significant for *L. guatemalensis*. Our findings are limited to the acute inflammation study conducted in male CD1 mice and cannot be extrapolated to other conditions. Additional studies must validate this anti-inflammatory effect under chronic administration conditions, across different sexes, and in models of immune-mediated inflammation. Possibly, the Aztecs may have used these plant leaves indiscriminately to reduce swelling, as described in the de la Cruz-Badiano Codex. Although they may not have been distinguishable, the chemotaxonomy of both species suggests that they could exhibit qualitatively similar therapeutic activities when used. The human tolerability of these plants is empirically supported by their long-standing traditional use, particularly as a condiment, indicating general safety. However, it does not replace the need for detailed studies to assess their effectiveness and safety profiles accurately. The present research presents only preliminary results that pave the way for future investigations across various areas, including pharmacology and toxicology. This perspective will allow us to achieve greater success in translating our findings into clinical trials.

## 4. Materials and Methods

### 4.1. Plant Collection

Leaves of *L. glaucescens* were collected on 8 August 2021, in the town of Tomatlán, Zacatlán, Puebla, México (19°53′57.39″ N 97°57′52.095″ W). Leaves of *L. guatemalensis* and the herbarium specimen were also collected on 21 October 2020, in the town of Tepoztlán, Morelos, México (18°59′59.6″ N 99°05′7.728″ W) (Figure S3). The herbarium specimens were deposited in the Faculty’s herbarium of Sciences, UNAM (FCME) under numbers 186887 for *L. glaucescens* and 179248 for *L. guatemalensis*. Figure S4: The collected leaves were dried at room temperature under artificial light. For more details, see Figures S5 and S6.

### 4.2. Preparation of Plant Extracts

The dried leaves of *L. glaucescens* (235.05 g) and *L. guatemalensis* (39.30 g) were separately macerated in methanol (MeOH). The resulting extracts were then concentrated under reduced pressure with a rotary evaporator (Büchi Rotavapor® RE-111 with Water Bath B-461, Büchi Labortechnik AG, Flawil, Switzerland). The dried methanolic extracts GLAM and GUAM were washed with hexane, yielding the hexane-soluble fractions GLAHF and GUAHF, and the methanol-soluble fractions GLAMF and GUAMF.

### 4.3. Chemicals and Solutions

Ethyl acetate, hexane, methanol, toluene, and formic acid reagents were purchased from JT BakerTM (Phillipsburg, NJ, USA). HPLC-grade acetonitrile was obtained from LiChrosolv (Merck KGaA, Darmstadt, Germany). The reagents, including Hexadecyltrimethylammonium bromide, 3,3’,5,5’, Tetramethyl-benzidine, *N, N*-Dimethylformamide, TPA (12-tetradecanoylphorbol 13-acetate), DPPH (2,2-Diphenyl-1-picrylhydrazyl), carrageenan, β-ethylaminodiphenylboric acid, indomethacin, and Quercetin dihydrate standard were purchased from Sigma-Aldrich (St. Louis, MO, USA). Quercitrin and pinocembrin standard (Santa Cruz, CA, USA). Silica gel 60 M MACHEREY-NAGEL GmbH & Co. KG (Düren, Germany). The water was purified by a Milli-Q^®^ system from Millipore (Bedford, MA, USA). We filtered the solvents through a 0.45 µm membrane filter (GVS, Apodaca, NL, México) and through silica gel 60 M (MACHEY-NAGEL GmbH & Co. KG, Düren, Germany). Indomethacin, GLAM, and GUAM extracts, as well as GLAMF and GUAMF, were diluted with saline solution for in vivo assays. All extracts were free of methanol. Indomethacin was mixed with 0.5% (*w*/*v*) sodium bicarbonate.

### 4.4. Thin-Layer Chromatography (TLC)

The complete methanol extracts of both plants (GLAM and GUAM) and their non-polar fractions (GLAHF, GUAHF) and polar fractions (GLAMF, GUAMF) were compared using TLC of 0.20 mm silica gel 60, F254 plates (MACHEY-NAGEL GmbH & Co. KG, Düren, Germany). Two mobile phases were used: mobile Phase 1 included toluene-ethyl acetate-formic acid in a ratio of 6:3, and mobile phase 2 was prepared with ethyl acetate-water-formic acid in a ratio of 8:1:1. Based on the reported phytochemicals in the literature [19,20], the chromatographic zones were visualized using 254 nm and 365 nm UV light, as well as after derivatization with 1% 2-aminoethyl diphenylborinate in TLC analysis.

### 4.5. HPLC of GLAM

The HPLC method was used to characterize and quantify the secondary metabolites of the GLAM extract. An HPLC system from Waters (Milford, MA, USA), incorporating a 996 photodiode-array detector (DAD), a 717 plus auto-sampler, and a 600 pump, was used for the analysis. A 10 µL volume of the GLAM (10 mg/mL) sample was injected into an Agilent SB C18 Zorbax column (4.6 × 150 mm, 5 µm). The column temperature was 30 °C. The mobile phase used was 0.1% (*v*/*v*) formic acid in water (Phase A) and 0.1% (*v*/*v*) formic acid in acetonitrile (Phase B). The gradient program was 15% B (0–1 min), 35% B (1–20 min), 45% B (20–25 min), and 15% B (25–35 min), with a flow rate of 1 mL/min. The column eluate was monitored at 200–400 nm. Literature suggests that both species primarily contain flavonoids, which informs these methods. This procedure was performed in triplicate to obtain the retention time.

### 4.6. GLAMF Fractionation, HPLC, and MS

After washing the complete GLAM extract with hexane, the resulting product, GLAMF (yield 15.82 g), was fractionated. Celite was added to 50 mg of GLAMF dissolved in 3 mL of methanol, and the mixture was dried under a stream of N_2_. The resulting mixture was loaded into a solid-phase extraction (SPE) Bond Elut C18 cartridge (Agilent, Part number 12102028), which was then placed in an Alltech vacuum manifold for fractionation. The fractions were recovered under vacuum using a gradient from 100% water to 100% methanol (MeOH). This procedure generated nineteen fractions. Samples were analyzed at the chemistry institute’s chromatography unit using an Agilent 1260 Infinity II Liquid Chromatograph equipped with a diode-array UV detector. Chromatograms were recorded at wavelengths of 210, 280, and 340 nm. Separation was performed on a Luna 5 µm C18 (2) 100 Å, 250 × 4.6 mm column (Phenomenex).

The mobile phase consisted of acetonitrile (ACN) and water containing 0.1% formic acid. The chromatographic gradient began at 30:70 (ACN:H_2_O) and increased linearly to 100:0 over 20 min. The flow rate was set to 1.0 mL/min.

The fraction obtained from the 70:30% methanol-water gradient that showed the majority peak was analyzed by Direct Analysis in Real-Time Mass Spectrometry (DART MS) in positive-ion mode at the National Laboratory of Sciences for Research and Conservation of Cultural Heritage at the Institute of Chemistry, LANCIC-IQ UNAM, and in Proton nuclear magnetic resonance (^1^H NMR).

### 4.7. GLAHF Fractionation and GC-MS

A gas chromatograph coupled to a mass spectrometer (GC/MSD) 7890B-5977A (Agilent Technologies, Santa Clara, CA, USA) was used to identify the compounds present in GLAHF. Separation was performed on a 30 m long, 0.25 mm diameter, 0.25 µm film-thickness HP-5ms column (5% phenyl, methylpolysiloxane, J&W, Santa Clara, CA, USA). The injection port was set at 280 °C, and 1 µL of the extract was injected in split mode with a 20:1 ratio. Analyses were performed using the following temperature program: an initial temperature of 40 °C for 1 min, ramped to 310 °C at 8 °C/min, and held for 10 min. Helium (He) was used as the carrier gas at a flow rate of 1 mL/min. Detection was performed using a mass spectrometer (Agilent Technologies, Santa Clara, CA, USA) with electron impact ionization at 70 eV. The source and quadrupole temperatures were 230 °C and 150 °C, respectively. The transfer line between the spectrometer source and the chromatograph was set at 310 °C. Data acquisition was performed in SCAN mode over the *m*/*z* range 30–600. Compound identification was performed by comparing GC/MS mass spectra with those reported in the NIST 14 library, achieving a match factor greater than 80. The GLAHF was analyzed using GC-MS, and the resulting data were compared with the NIST database at the National Laboratory for Research and Conservation of Cultural Heritage (LANCIC-IQ UNAM).

### 4.8. 1NMR of GLAMF and GLAHF

1HNMR spectra were obtained using a 400 MHz JNM-ECZS NMR spectrometer from JEOL (Japan Electron Optics Laboratory, Tokyo, Japan). 5 mg of the sample, 70:30% methanol-water, obtained from GLAMF, and 5 mg of one fraction of GLAHF were dissolved in 0.5 mL of deuterated methanol in a glass NMR tube with tetramethylsilane (TMS) to calibrate the chemical shift in the sample signals. The system was calibrated using the deuterated solvent signal (lock), and the magnetic field homogeneity (shimming) was optimized.

### 4.9. Animal Care

The 8-week-old (25–30 g) male CD-1 strain mice and the 6-week-old (200–250 g) male Wistar strain rats were used for the experiments. Researchers kept the animals in transparent acrylic boxes at room temperature (21–23 °C) on a 12 h light:12 h dark cycle and fed them pellet food (5001 Rodent Laboratory Chow) ad libitum. Their care was in line with Mexican standards NOM-062-ZOO-1999 and NOM-087-SEMARNAT-SSA1-2002 [59,60], as well as the Guide for the Care and Use of Laboratory Animals, revised in 2011 [61].

### 4.10. Carrageenan-Induced Paw Inflammation

This experiment was conducted in accordance with the protocols established by Morris [62] and Posadas et al. [63]. Male CD-1 strain mice, 8-week-old (25–30 g), were randomly numbered and then assigned to one of the ten groups using a random number table. Ten groups, each with ten animals, received different treatments via intraperitoneal delivery. Group I served as the negative control and received only saline solution (0.9%). Group II served as a positive control and received indomethacin at 10 mg/kg, dissolved in distilled water. Groups III, IV, V, and VI received 10, 31, 100, and 310 mg/kg of GLAM extract, respectively. Groups VII, VIII, IX, and X received 310 mg/kg of GUAM extract, respectively. After a 60 min interval following the treatments, 0.05 mL of a 1% carrageenan solution was injected into the paw to induce inflammation. The thickness of the paw was measured using a digital micrometer (0–25 mm 0.001 mm Metric) (Fujisan) before the administration of the extracts (baseline) and after the administration of carrageenan at 1, 2, 3, 4, 5, and 24 h post-treatment. The edema was calculated using this formula: Edema (%) = (M_f_ − M_i_/M_i_) (100). Where M_f_ is the paw measurement at different times, and M_i_ is the paw measurement before inflammation was induced.

### 4.11. TPA-Induced Mouse Ear Inflammation

Male CD-1 strain mice, weighing 25 to 30 g, were enumerated and randomly assigned through a random number table to four treatment groups, each comprising seven animals. Under general anesthesia with Ketamine + Xilacine (65 mg/kg + 13 mg/kg administered intraperitoneally using a 27 G needle), 10 µL of an ethanolic solution of TPA at a concentration of 0.25 mg/mL was topically applied to the right ear of each mouse. Ten minutes later, the treatments were also applied to the right ear. Group I served as the negative control, while Group II acted as the positive control, receiving 20 µL of indomethacin (1 mg per ear). Group III received 20 µL of GLAM extract, and Group IV received 20 µL of GUAM extract, both at a concentration of 1 mg per ear. Four hours after the application of TPA, the animals were euthanized using chamber CO_2_, and a 7 mm diameter biopsy was taken from both ears. The biopsies were weighed, and the difference in weight between the right and left biopsies was used to measure the edema. The mean weight differences in each treated group were compared to those of the control group, which received TPA and saline solution. The left ear of all groups did not receive TPA; instead, it received the vehicle’s ethanol (from TPA) and methanol (from the samples). The weight of the left ear contrasts with the increase in the right ear’s weight resulting from TPA application.

### 4.12. MPO Activity

MPO activity in tissue was measured across all four treatment groups using a modified method based on the protocols by Bradley et al. [64] and Suzuki et al. [65]. The mice were enumerated and randomly assigned to four treatment groups using a random number generator, with five animals per group. Biopsies taken from mouse ears four hours after TPA administration were placed in 1 mL of 80 mM phosphate-buffered saline (PBS) at pH 5.4, containing 0.5% hexadecyltrimethylammonium bromide (HTAB). The sample was homogenized for 30 s at 4 °C with a Tissue Homogenizer (OMNI International, model 125, Kennesaw, Georgia, USA). The homogenate was then subjected to three freeze–thaw cycles at room temperature, followed by sonication for 20 s and centrifugation at 12,000 rpm for 15 min at 4 °C in an Eppendorf 5415 R (Hamburgo, Alemania). From the resulting supernatant, quadruplicate 10 μL aliquots were placed into a 96-well microplate, followed by the addition of 180 μL of 80 mM PBS (pH 5.4) without HTAB. The microplate was heated to 37 °C, after which 20 μL of 0.017% hydrogen peroxide was added to each well. To start the MPO activity reaction, 20 μL of 18.4 mM 3,3′,5,5′-tetramethylbenzidine (TMB) dissolved in 50% aqueous dimethylformamide (DMF) was added. The microtiter plates were incubated at 37 °C for 5 min, and the reaction was stopped by adding 20 μL of 2 M sulfuric acid (H_2_SO_4_). MPO enzyme activity was evaluated colorimetrically using a BioTek Microplate Reader (SYNERGY HT) at a wavelength of 450 nm. MPO activity was expressed as optical density (OD) per biopsy.

### 4.13. DPPH Free Radical Scavenging Activity

The antioxidant activity of GLAM and GUAM complete extracts, as well as the GLAMF and GUAMF fractions, was assessed at concentrations of 5.62, 10, 17.78, 31.62, 56.23, and 100 µg/mL using the DPPH decolorization model. This procedure was based on the method described by Domínguez et al. [66], with modifications specific to this application.

To perform the assay, first prepare the test solution at various concentrations; pipette 50 mL of each into a 96-well microplate in triplicate. Then, add 150 mL of a 133.33 mM ethanolic DPPH solution to each well, bringing the final concentration to 100 mM. The microplate was incubated at 37 °C for 30 min in the dark with shaking. After incubation, the absorbance of each well was measured at 515 nm using a BioTek Instruments (Winooski, VT, USA) Microplate Reader (SYNERGY HT).

Each sample was analyzed in triplicate, and the results were presented as the mean of three independent experiments, with the standard error of the mean. The antioxidant activity against DPPH is expressed as a percentage of reduction, calculated using the formula: DPPH reduction (%) = (C − E)/C (100), where C represents the optical density (OD) of 100 µM DPPH, and E represents the OD of the 100 µM DPPH + sample mixture. The antioxidant potential of the methanolic extracts of the *Litsea* spp. was evaluated using a quercetin dihydrate standard at concentrations of 1.07, 1.90, 3.38, 6.01, and 10.69 µg/mL. The 50% inhibitory concentration (IC50) is the concentration of the plant extract required to inhibit 50% of MPO activity. Lower IC_50_ values indicate greater enzyme inhibitory activity.

### 4.14. TBARS Assay for Lipid Oxidation

The antioxidant activity of GLAM and GUAM extracts against oxidative stress in the entire brains of adult male Wistar rats (200–250 g) was evaluated using the TBARS assay, performed according to Ohkawa et al. [67] with adjustments. Following homogenization in phosphate-buffered saline (PBS), the sample was centrifuged at 3000 rpm for 10 min; the pellet was subsequently removed. Protein quantification in the supernatant was performed using the Lowry et al. [68] method, and the concentration was adjusted to 2.666 mg/mL with PBS. Stock solutions of GLAM and GUAM extracts were prepared with distilled water, yielding six concentrations with a quarter-log progression (5.62, 10, 17.78, 31.62, and 56.23 (µg/mL). Into each chilled micro-centrifuge tube, 25 µL of each extract solution was added, followed by 375 µL of supernatant (corresponding to 1 mg of protein) and 50 µL of EDTA (20 µM in saline). The process began when 50 µL of a 100 µM FeSO_4_ solution was added to each tube, and then the tubes were incubated at 37 °C for 1 h to start lipid peroxidation. Subsequently, TBARS content was quantified by mixing 0.5 mL of a 0.5% thiobarbituric acid reagent in 0.05 N NaOH with 30% trichloroacetic acid at a 1:1 volume ratio. The final suspension was refrigerated on ice for 10 min, then centrifuged at 12,000 rpm for 5 min, and finally heated in a 75 °C water bath for 30 min. Once the sample reached room temperature, its absorbance was measured at 540 nm using a BioTek Instruments (Winooski, VT, USA) Microplate Reader (SYNERGY HT).

Concentration of TBARS was calculated by interpolation in a standard curve of tetramethoxypropane [69]. Results were expressed as nmol of TBARS per mg of protein. Quercetin was used as a positive standard at the following concentrations: 0.108, 0.189, 0.338, 0.602, 1.069, 1.901, and 3.383 µg/mL. The formula RI = (C − E)/C (100) was used to determine the inhibition index (RI [%]), where C and E denote the absorbance of the control and sample, respectively. These values were plotted against the logarithm of each extract’s concentration, and an EC ≤ 50 was defined as the concentration required to achieve a 50% reduction in peroxidation.

### 4.15. Statistics

In the carrageenan model, the data were analyzed by comparing the averages of baseline measurements (taken before treatment) to the values recorded at each designated time for each group, using a two-way analysis of variance (ANOVA), followed by Dunnett’s multiple-comparison test to identify statistically significant differences between groups (*p* ≤ 0.05). The examination of the TPA and MPO models centered on the mean weight disparity between the right (treated) and left (untreated) ears. Within the myeloperoxidase model, the mean optical density from biopsies was computed for each group. A one-way analysis of variance (ANOVA) was performed, followed by Dunnett’s multiple-comparison test to identify statistically significant differences between groups (*p* ≤ 0.05). To determine IC_50_ values, we used a 4-parameter logistic (4PL) regression model for the DPPH and TBARS assays. GraphPad Prism 8.4.3 (GraphPad Software, Inc., San Diego, CA, USA) was used for statistical analysis and graphics. The parameters obtained from this model are reported in Table S4. The values in each experimental model are presented as the mean ± standard error of the mean.

## 5. Conclusions

This research, for the first time, shows that using an extract from *L. glaucescens* leaves, either topically or systemically, reduces neutrophil activity and oxidative damage in vitro, and decreases swelling in vivo. The anti-inflammatory activity of the methanolic extract of *L. glaucescens* leaves is significantly greater than the activity observed with the extract of *L. guatemalensis*, especially when the extracts are administered systemically in vivo. Quercitrin is the main component in the polar part of this extract of *L. glaucescens* leaves, while pinocembrin is in the less polar part. Our findings, in conjunction with previous reports on these two phytochemicals, suggest that these substances are involved in signaling pathways related to inflammatory processes and may contribute to the anti-inflammatory effects of *L. glaucescens* leaves. However, further experimental validation is required. We do not know which plant, *L. glaucescens* or *L. guatemalensis*, the Aztecs used. However, our findings show that both plants reduce inflammation. This suggests that the Aztecs were aware of the medicinal benefits of these plants.

## Figures and Tables

**Table 1:** Ear weight without and with edema produced by TPA administration. Summarization of the raw weight values of the left (without TPA) and right ears (with TPA) of mice, plus the treatments.

Treatment	$\bar{x}$ of Ears Left, Weight ± SEM (mg) Without TPA	$\bar{x}$ of Ears Right, Weight ± SEM (mg) with TPA
Ethanolic solution	13.7 ± 0.6	28.6 ± 0.7
Indomethacin (1 mg/ear)	12.8 ± 0.6	18.4 ± 2.0
GLAM (1 mg/ear)	11.4 ± 0.2	22.0 ± 1.3
GUAM (1 mg/ear)	13.3 ± 0.8	25.5 ± 0.9

GLAM: methanolic extract of *L. glaucescens*, GUAM: methanolic extract of *L. guatemalensis*, TPA: 12-tetradecanoylphorbol 13-acetate, $\bar{x}$: mean ± SEM: standard error of the mean, n = 9 animals per group.

**Table 2:** Inhibitory effect of the extracts from GLAM and GUAM leaves on myeloperoxidase activity in the right ears of the mouse. The right-hand column summarizes the optical density (OD) values for each treatment.

Treatment	$\mathrm{OD}\;\bar{x}$ ± SEM
Ethanolic solution (basal)	0.08 ± 0.01
Ethanolic solution + TPA	1.25 ± 0.19
Indomethacin (1 mg/ear) + TPA	0.33 ± 0.19
GLAM (1 mg/ear) + TPA	0.31 ± 0.11
GUAM (1 mg/ear) + TPA	0.15 ± 0.02

GLAM: methanolic extract of *L. glaucescens*, GUAM: methanolic extract of *L. guatemalensis*, OD: optical density; $\bar{x}$: mean ± SEM: standard error of the mean, n: 5 animals per group.

**Table 3:** Inhibitory effects of GLAM and GUAM leaf extracts on the DPPH radical scavenging capacity in vitro. A summary of the raw absorbance values for each concentration and methanolic extract is presented.

Concentration (µg/mL)	$\mathrm{Absorbance}\;\bar{x}$ ± SEM	
	GLAM	GUAM
0	0.64 ± 0.003	0.64 ± 0.003
5.62	0.57 ± 0.002	0.57 ± 0.001
10	0.52 ± 0.002	0.51 ± 0.002
17.78	0.43 ± 0.001	0.42 ± 0.001
31.62	0.30 ± 0.004	0.28 ± 0.002
56.23	0.1 ± 0.004	0.1 ± 0.002
100	0.0 ± 0.0007	0.0 ± 0.001

GLAM: methanolic extract of *L. glaucescens*, GUAM: methanolic extract of *L. guatemalensis*, DPPH: 2,2-Diphenyl-1-picrylhydrazyl, Absorbance $\bar{x}$: mean ± SEM: standard error of the mean.

**Table 4:** Inhibitory effect of GLAM and GUAM leaves on the levels of TBARS—summarization of the raw absorbance values for each treatment.

Concentration (µg/mL)	$\mathrm{nmol}/\mathrm{mg}\;\mathrm{Prot}.\;\bar{x}$ ± SEM	
	GLAM	GUAM
Basal	0.70 ± 0.25	0.70 ± 0.25
FeSO_4_	10.53 ± 0.47	10.53 ± 0.47
5.62	9.22 ± 0.45	9.16 ± 0.53
10	8.81 ± 0.49	8.85 ± 0.45
17.78	7.69 ± 0.39	7.67 ± 0.46
31.62	5.62 ± 0.33	6.07 ± 0.41
56.23	2.05 ± 0.37	2.28 ± 0.68

FeSO_4_: ferrous sulfate. GLAM: methanolic extract of *L. glaucescens*, GUAM: methanolic extract of *L. guatemalensis*, $\bar{x}$: mean, SEM: standard error of the mean, n: 5 animals per group.

## Data Availability

The original contributions presented in this study are included in the article/Supplementary Materials. Further inquiries can be directed to the corresponding authors.

## Supplementary Materials

The following supporting information can be downloaded at https://www.mdpi.com/article/10.3390/ph19010040/s1: Figure S1. TLC of extracts and fractions from *L. glaucescens* (A and C) and *L. guatemalensis* (B and D). The samples were eluted using two different solvent systems with varying polarities: toluene-ethyl acetate-formic acid in a 6:3:1 ratio (A and B) and ethyl acetate-water-formic acid in an 8:1:1 ratio (C and D). Refer to the abbreviations for the meanings of the acronyms. Figure S2. UV spectra obtained from 200 to 400 nm show the absorbance peaks of the quercetin and pinocembrin standards present in GLAM extract. Figure S3. UV absorbance from 200 to 400 nm with respect to Quercitrin and Pinocembrin standards, and their presence in the whole extracts of GLAM. (A) Quercitrin standard spectra, (B) Pinocembrin standard spectra, (C) Quercitrin spectra from GLAM extract, and (D) Pinocembrin spectra from GLAM extract. The pattern of the peaks from A-D indicates that the presence of both Quercitrin and Pinocembrin can be observed in the GLAM extract. Figure S4. HPLC chromatograms of different GLAMF fractions obtained on a SPE cartridge with water-methanol mixtures of 70:30 (A) at 280 nm, and (B) at 340 nm. Figure S5. *L. glaucescens* collected in Tomatlán, Zacatlán, Puebla. Mexico. *L. guatemalensis* collected in Tepoztlán, Morelos, Mexico. Figure S6. (A) Herbarium specimen of *L. glaucescens* with the number 186887. (B) Herbarium specimen of *L. guatemalensis* with the number 179248. Table S1. Summary of the compounds identified in the hexane fractions from *L. glaucescens* using NIST database. Table S2. Inhibitory effect of GLAM leaf extracts on Carrageenan-induced paw inflammation (mm). Table S3. Inhibitory effect of GUAM leaf extracts on Carrageenan-induced paw inflammation (mm). Table S4. Inhibitory effect of GLAM and GUAM leaf extracts on TPA-induced ear inflammation. Summarization of the weight of the left (without TPA) and right ears (with TPA) of mice. Table S5. Inhibitory effect of GLAM and GUAM leaves on myeloperoxidase (MPO) activity in mouse ears. Summarization of the optical density (OD) values for each treatment.
