# Supplementary material for: Anti-Inflammatory and Antioxidant Activity of Litsea glaucescens Kunth in Rodents, an Aztec Medicinal Plant Used in Pre-Columbian Times
Source: Pharmaceuticals (Basel). 2025 Dec 23;19(1):40. doi: 10.3390/ph19010040 (PMC12844792; doi:10.3390/ph19010040)
Supplement: Supplementary file 1 [file pharmaceuticals-19-00040-s001.zip › pharmaceuticals-4028484-supplementary.pdf]

SUPPLEMENTARY DATA

Anti-inflammatory and antioxidant activity of *L. glaucescens* Kunth in rodents, an Aztec medicinal plant used in pre-Columbian times

Dulce Yehimi López-Miranda [1,2], Ricardo Reyes-Chilpa*[3], Antonio Nieto-Camacho[3], Silvia Laura Guzmán Gutiérrez, Oscar Salvador Barrera-Vázquez[1], Juan Luis Escobar-Ramírez[1], and Gil Alfonso Magos-Guerrero[1]*

[1]Universidad Nacional Autónoma de México, Facultad de Medicina, Copilco Universidad, Coyoacán, 04360 Cd. Mx., México
[2]Universidad Nacional Autónoma de México, Posgrado en Ciencias Biológicas, 04510 Cd. Mx., México.
[3]Universidad Nacional Autónoma de México, Instituto de Química, Ciudad Universitaria, 04510 Cd. Mx., México
*Correspondence: gamagos@unam.mx Tel.: 52+55 5512 869955 (GAMG), chilpa@unam.mx.

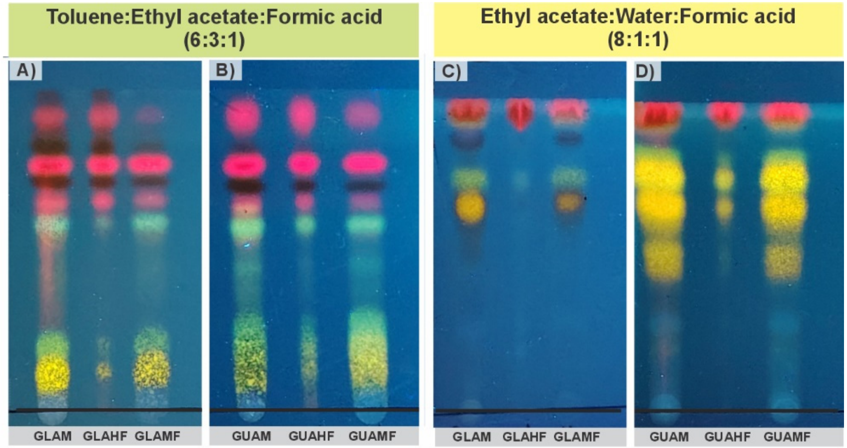

Figure S1. TLC of extracts and fractions from *L. glaucescens* (A and C) and *L. guatemalensis* (B and D). The samples were eluted using two different solvent systems with varying polarities: toluene-ethyl acetate-formic acid in a 6:3:1 ratio (A and B) and ethyl acetate-water-formic acid in an 8:1:1 ratio (C and D). Refer to the abbreviations for the meanings of the acronyms.

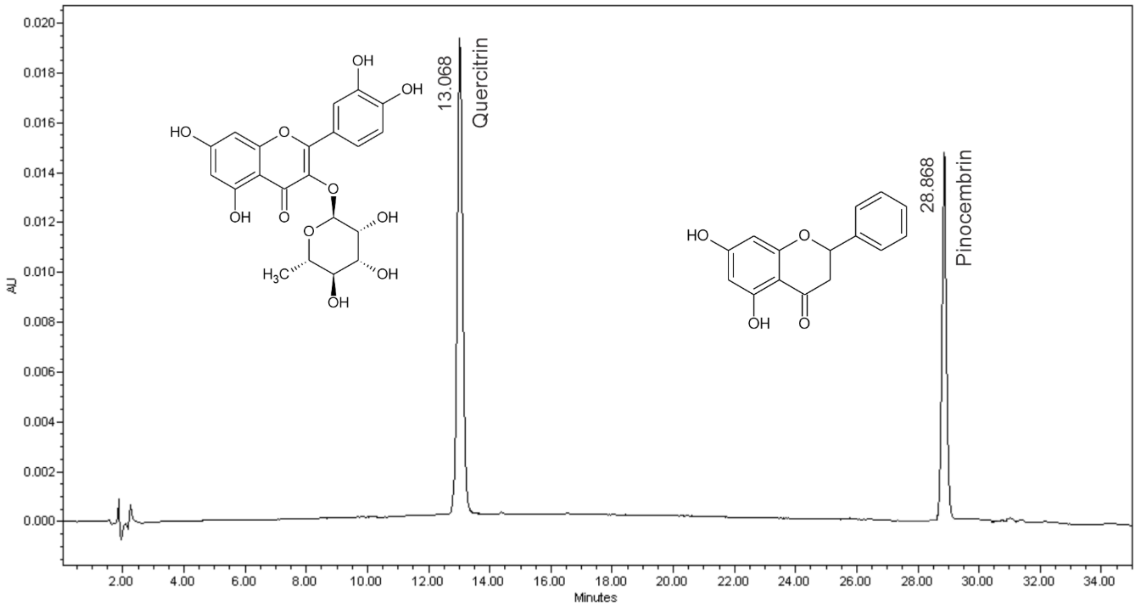

Figure S2. UV spectra obtained from 200 to 400 nm show the absorbance peaks of the quercetin and pinocembrin standards present in GLAM extract.

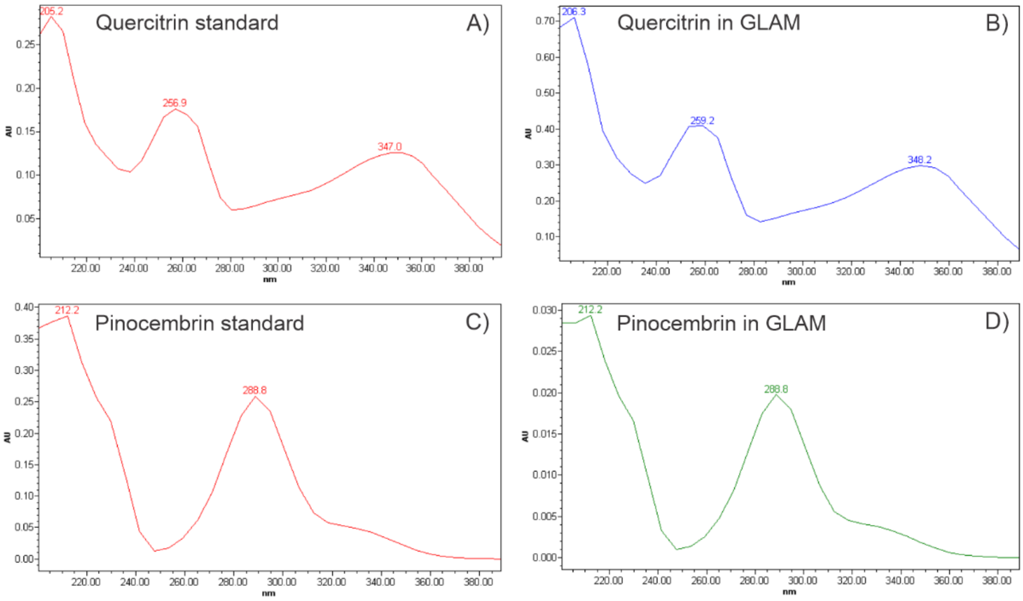

Figure S3. UV absorbance from 200-400 nm with respect to Quercitrin and Pinocembrin standards, and their presence in the whole extracts of GLAM. A) Quercitrin standard spectra, B) Pinocembrin standard spectra, C) Quercitrin spectra from GLAM extract, and D) Pinocembrin spectra from GLAM

extract. The pattern of the peaks from A-D indicates that the presence of both Quercitrin and Pinocembrin can be observed in the GLAM extract.

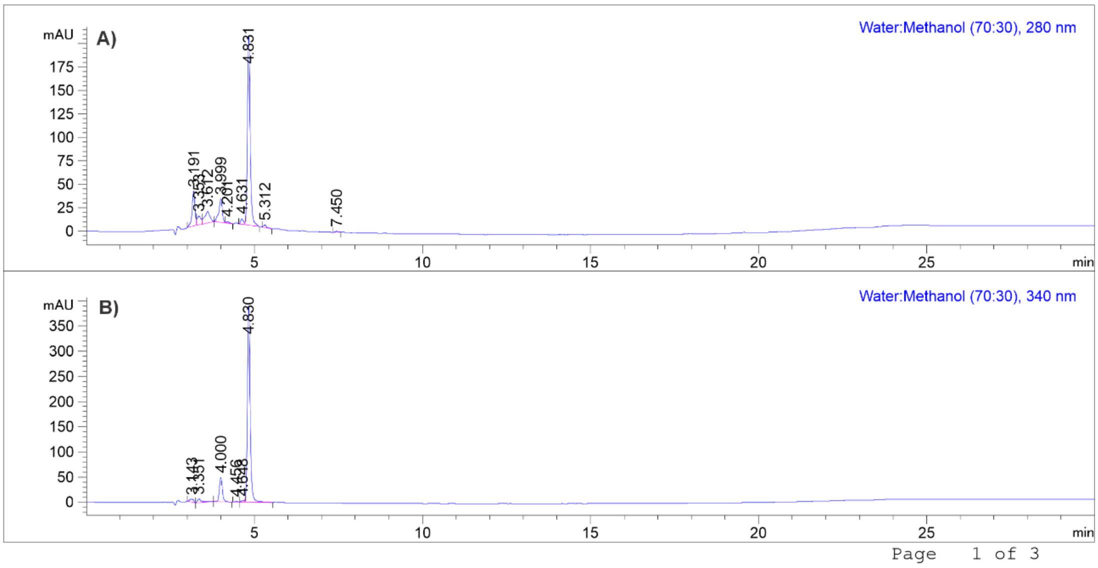

Figure S4. HPLC chromatograms of different GLAMF fractions obtained on a SPE cartridge with water-methanol mixtures of 70:30 (A) at 280 nm, and (B) at 340 nm.

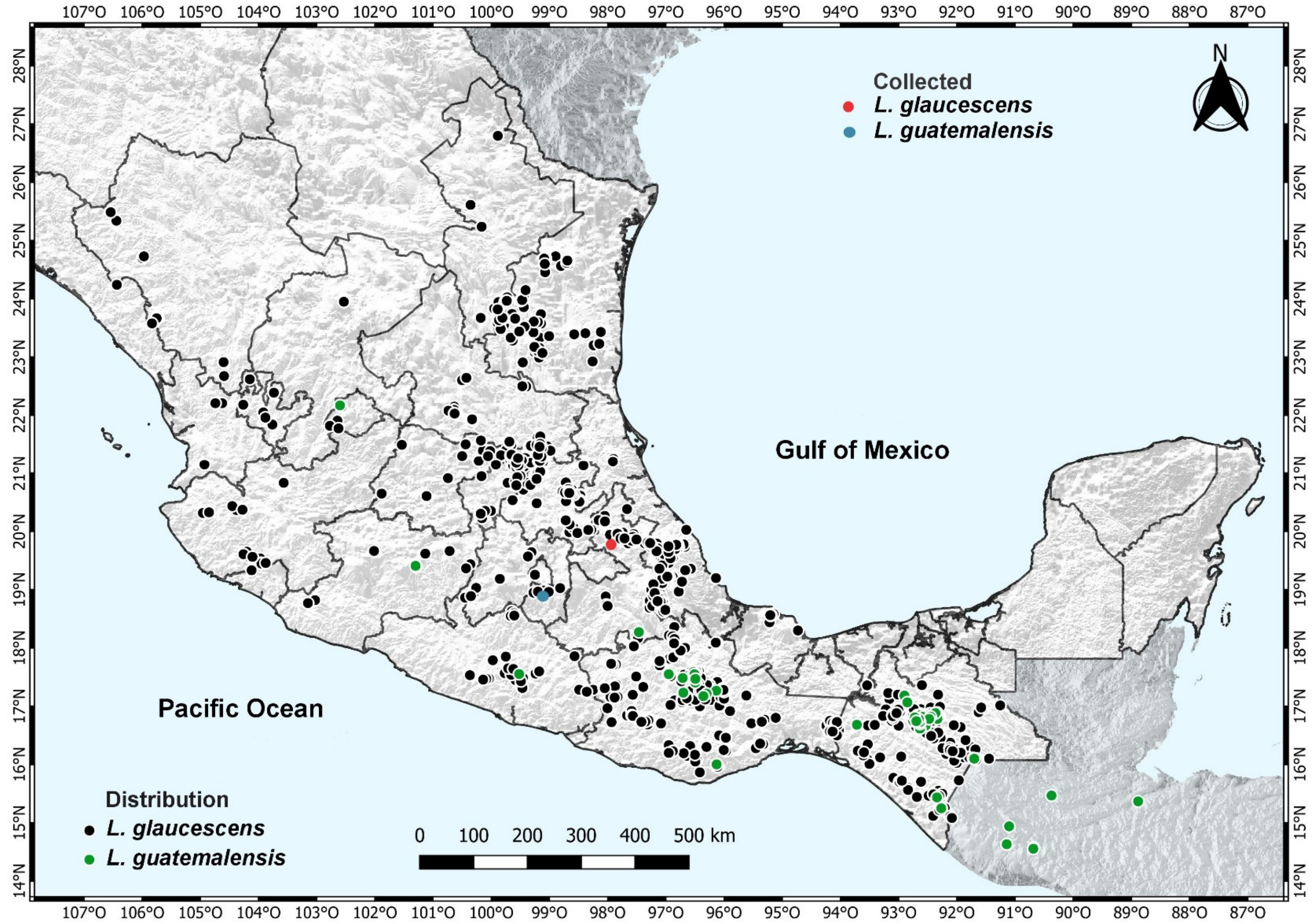

Figure S5. 🔴 *Litsea glaucescens* collected in Tomatlán, Zacatlán, Puebla. Mexico. 🔵 *Litsea guatemalensis* collected in Tepoztlán, Morelos, Mexico.

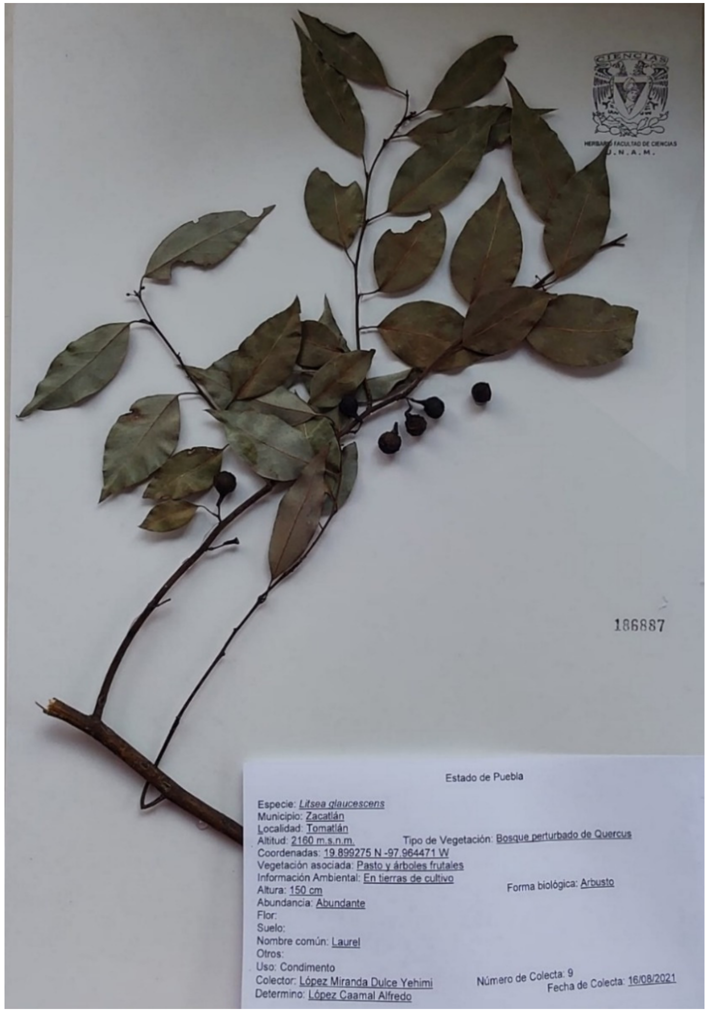

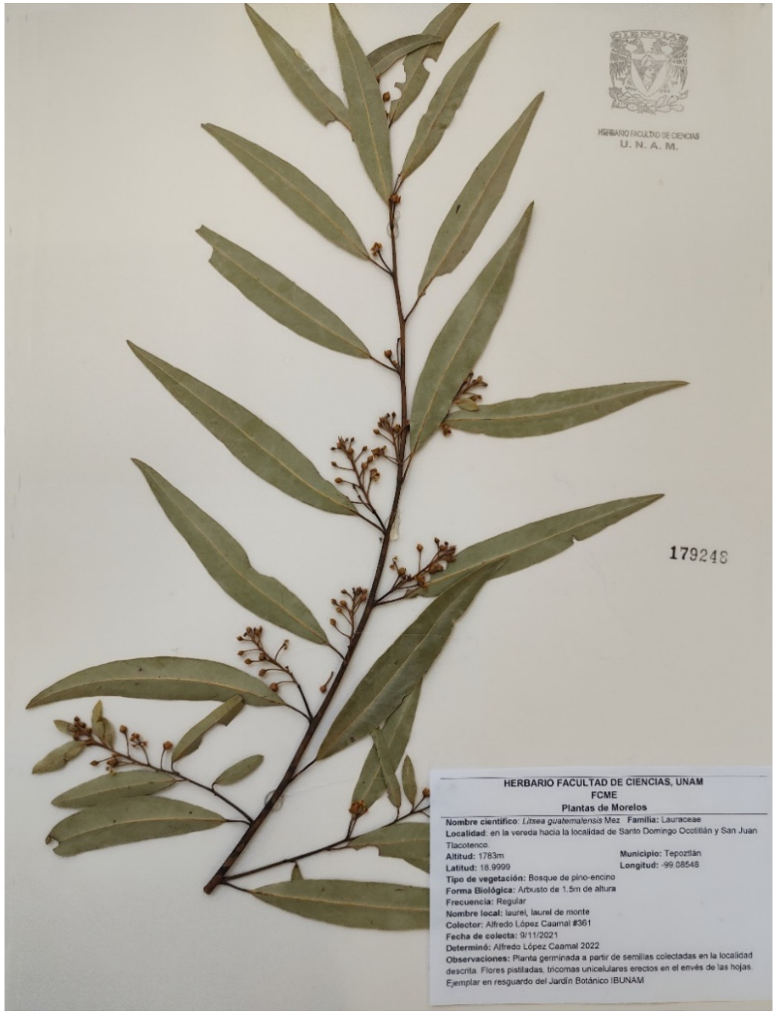

Figure. S6. A) Herbarium specimen of Litsea glaucescens with the number 186887. B) Herbarium specimen of Litsea guatemalensis with the number 179248

Table S1. Summary of the compounds identified in the hexane fractions from *L. glaucescens* using NIST database.

No	Name	Molecular Formula	RT	Relative Contents (%)	Match
1	ND	-	8.34	1.37	-
2	Ethane, hexachloro-	C_2Cl_6	9.27	10.05	90.3
3	Ethanone, 1-(4-methylphenyl)-	$C_9H_{10}O$	11.36	1.80	82.5
4	NI	-	11.74	1.23	-
5	NI	-	13.90	2.35	-
6	NI	-	14.36	2.15	-
7	NI	-	15.40	2.00	-
8	NI	-	15.94	1.56	-
9	NI	-	16.21	2.08	-
10	NI	-	16.88	3.33	-
11	Dodecanoic acid	$C_{12}H_{24}O_2$	17.46	1.22	76.5
12	NI	-	18.08	1.49	-
13	NI	-	18.33	2.10	-
14	Tetradecanoic acid	$C_{14}H_{28}O_2$	20.26	1.70	77.9
15	Phytol, acetate	$C_{22}H_{42}O_2$	21.33	4.52	76.1
16	2-Pentadecanone, 6,10,14-trimethyl-	$C_{18}H_{36}O$	21.41	1.68	89.6
17	NI	-	21.87	1.93	-
18	NI	-	22.38	1.89	-
19	n-Hexadecanoic acid	$C_{16}H_{32}O_2$	22.84	5.99	88.2
20	NI	-	25.00	2.05	-
21	Pinocembrin	$C_{15}H_{12}O_4$	28.66	17.08	96.0
22	NI	-	32.24	2.24	-
23	NI	-	34.00	11.38	-
24	NI	-	34.16	2.47	-
25	NI	-	34.25	1.75	-
26	dl-alpha-Tocopherol	$C_{29}H_{50}O_2$	34.38	2.67	83.7
27	NI	-	35.48	1.40	-
28	NI	-	36.03	2.19	-
29	NI	-	36.12	6.31	-
			Total contents of compounds	100	

NI=Not identified.

Table S2. Inhibitory effect of GLAM leaf extracts on Carrageenan-induced paw inflammation (mm).

Treatment	Inflammation (mm)						
	Time (h)						
	0	1	2	3	4	5	24
Carrageenan (50 μg/paw)	3.7 ± 0.1	4.7 ± 0.1	4.9 ± 0.1	5.1 ± 0.1	5.1 ± 0.1	5.0 ± 0.1	4.2 ± 0.3
Indomethacin (10 mg/Kg)	3.1 ± 0.1	4.2 ± 0.1^{ns} p = 0.0750	4.4 ± 0.1* p = 0.0150	4.6 ± 0.2 p = 0.0780	4.5 ± 0.1* p = 0.0048	4.5 ± 0.2* p = 0.0236	3.4 ± 0.2*** p = 0.0002
GLAM (310 mg/Kg)	3.2±0.02	4.1 ± 0.1*** p = 0.0004	4.1 ± 0.1**** p = <0.0001	4.1 ± 0.1**** p = <0.0001	4.3 ± 0.1**** p = <0.0001	4.2 ±0.04**** p = <0.0001	3.6 ± 0.05*** p = 0.0002
GLAM (100 mg/Kg)	3.4±0.05	4.1 ± 0.1**** p = <0.0001	4.1 ± 0.2**** p = <0.0001	4.2 ± 0.1**** p = <0.0001	4.3 ± 0.2**** p = <0.0001	4.3± 0.03**** p = <0.0001	3.9 ± 0.05** p = 0.0054
GLAM (31 mg/Kg)	3.3±0.1	4.3 ± 0.1** p = 0.0094	4.5 ± 0.04** p = 0.0038	4.6 ± 0.1** p = 0.0028	4.4 ± 0.1**** p = <0.0001	4.4 ± 0.1*** p = 0.0003	4.0 ± 0.1^{ns} p = 0.0839
GLAM (10 mg/Kg)	3.2±0.01	4.7 ± 0.05^{ns} p = 0.5727	5.1 ± 0.1^{ns} p = 0.9938	5.3 ± 0.04^{ns} p = 0.9985	5.2 ± 0.1^{ns} p = 0.7393	5.2 ± 0.04^{ns} p = 0.9856	4.4 ± 0.004^{ns} p = >0.9999

Two-way ANOVA Dunnett's post hoc test.

ns = p > 0.05 (non-significant), *p ≤ 0.05, **p ≤ 0,01, ***p ≤ 0,001, ****p ≤ 0,0001.

Table S3. Inhibitory effect of GUAM leaf extracts on Carrageenan-induced paw inflammation (mm).

Treatment	Inflammation (mm)						
	Time (h)						
	0	1	2	3	4	5	24
Carrageenan (50 μg/paw)	3.7 ± 0.1	4.7 ± 0.1	4.9 ± 0.1	5.1 ± 0.1	5.1 ± 0.1	5.0 ± 0.1	4.2 ± 0.3
Indomethacin (10 mg/Kg)	3.1 ± 0.1	4.2 ± 0.1^{ns} p = 0.0750	4.4± 0.1* p = 0.0150	4.6 ± 0.2 p = 0.0780	4.5 ± 0.1* p = 0.0048	4.5 ± 0.2* p = 0.0236	3.4 ± 0.2*** p = 0.0002
GUAM (31 0mg/Kg)	3.1±0.1	4.3 ± 0.1** p = 0.0025	4.4 ± 0.1** p = 0.0016	4.6 ± 0.1** p = 0.0043	4.6 ± 0.1*** p = 0.0002	4.5 ± 0.1* p = 0.0313	4.1 ± 0.2^{ns} p = 0.9443
GUAM (100 mg/Kg)	3.3±0.03	4.4 ± 0.05** p = 0.0033	4.6 ± 0.1** p = 0.0044	4.9 ± 0.1* p = 0.0122	4.7 ± 0.1*** p = 0.0002	4.8 ± 0.1** p = 0.0091	4.50 ± 0.1^{ns} p = 0.9989
GUAM (31 mg/Kg)	3.3±0.03	4.3 ± 0.1* p = 0.0181	4.7 ± 0.03^{ns} p = 0.0603	4.7 ± 0.1* p = 0.0124	4.6 ± 0.1** p = 0.0023	4.7 ± 0.1* p = 0.0369	4.3 ± 0.1^{ns} p = 0.8267
GUAM (10 mg/Kg)	3.3±0.02	4.4 ± 0.1^{ns} p = 0.055	4.9 ± 0.1^{ns} p = 0.511	5.1 ± 0.04^{ns} p = 0.5192	5.2 ± 0.1^{ns} p = 0.5673	5.2 ± 0.03^{ns} p = 0.9641	4.4 ± 0.1^{ns} p = 0.9828

Two-way ANOVA Dunnett's post hoc test.

ns = p > 0.05 (non-significant), *p ≤ 0.05, **p ≤ 0,01, ***p ≤ 0,001, ****p ≤ 0,0001.

Table S4. Inhibitory effect of GLAM and GUAM leaf extracts on TPA-induced ear inflammation. Summarization of the weight of the left (without TPA) and right ears (with TPA) of mice.

Treatment	Ear Edema		% of inhibition of edema
	Without TPA (mg $\bar{x}$ ± SEM)	With TPA (mg $\bar{x}$ ± SEM)	
Normal control	13.7 ± 0.58	28.6 ± 0.69	0 ± 3.93
Indomethacin (1 mg/ear)	12.8 ± 0.57	18.4±2.00**** p = <0.0001	62.62 ± 9.28
GLAM (1 mg/ear)	11.4 ± 0.19	22.0 ± 1.27* p = 0.0203	29.13 ± 8.47
GUAM (1 mg/ear)	13.3 ± 0.77	25.5 ± 0.90^{ns} p = 0.1736	18.26 ± 4.01

One-way ANOVA Dunnett's post hoc test.
ns = $p > 0.05$ (non-significant), *$p \leq 0.05$, ****$p \leq 0{,}0001$.

Table S5. Inhibitory effect of GLAM and GUAM leaves on myeloperoxidase (MPO) activity in mouse ears. Summarization of the optical density (OD) values for each treatment.

Treatment	OD $\bar{x}$ ± SEM.	% inhibition of MPO activity $\bar{x}$ ± SEM.
Normal control	1.25 ± 0.19	0 ± 0.7
Indomethacin (1 mg/ear)	0.33 ± 0.19*** p = 0.0004	73.4 ± 15.34
GLAM (1 mg/ear)	0.31 ± 0.11*** p = 0.0002	75.2 ± 9.09
GUAM (1mg/ear)	0.15 ± 0.02**** p = <0.0001	88..3 ± 1.73

One-way ANOVA Dunnett's post hoc test.
***$p \leq 0{,}001$, ****$p \leq 0{,}0001$.
